# Tissue-Engineered Larynx: Future Applications in Laryngeal Cancer

**DOI:** 10.1007/s40136-017-0144-6

**Published:** 2017-03-14

**Authors:** Nick J. I. Hamilton, Martin A. Birchall

**Affiliations:** University College London Ear Institute, Gray’s Inn Road, London, UK

**Keywords:** Tissue engineering, Larynx, Regenerative medicine, Laryngeal cancer, Larynx transplant

## Abstract

**Purpose of Review:**

This article reviews the latest developments in tissue engineering for the larynx with a specific focus on the treatment of laryngeal cancer.

**Recent Findings:**

Challenges in tissue engineering a total larynx can be divided into scaffold design, methods of re-mucosalization, and how to restore laryngeal function. The literature described a range of methods to deliver a laryngeal scaffold including examples of synthetic, biomimetic, and biological scaffolds. Methods to regenerate laryngeal mucosa can be divided into examples that use a biological dressing and those that engineer a new mucosal layer de novo. Studies aiming to restore laryngeal function have been reported, but to date, the optimum method for achieving this as part of a total laryngeal transplant is yet to be determined.

**Summary:**

There is great potential for tissue engineering to improve the treatments available for laryngeal cancer within the next 10 years. A number of challenges exist however and advances in restoring function must keep pace with developments in scaffold design.

## Introduction

Laryngeal cancer is the second most common airway cancer after lung cancer with an estimated global incidence of 157,000 per annum [1]. Within the USA, an estimated 13,430 new cases have been diagnosed in 2016 with an estimated annual mortality of 3620 [2]. Conventional treatments for advanced laryngeal cancer can be divided into surgical and organ preserving treatments. Surgical therapies involve either a total or partial laryngectomies. Total laryngectomy involves the complete resection of the larynx with the creation of a tracheostome to create a new airway and a neo-pharynx to preserve swallowing. Whilst this offers the chance for wide surgical margins and good swallow function [3, 4], voice rehabilitation is variable and there are additional psychological and social impacts of having a permanent tracheostome that impact on the quality of life [5, 6]. Partial laryngectomy encompasses procedures involving a sub-total resection of the larynx to preserve function whilst aiming to achieve similar rates of cure as total laryngectomy [7]. Function and quality of life assessments have been demonstrated to be higher with this approach compared to total laryngectomy although the subset of patients in which this resection is possible is small and problems with aspiration remain [8–10]. Organ preserving therapies involve chemo-radiation with curative intent whilst leaving the anatomical structures of the larynx to preserve function. Survival rates have been shown to be comparable to surgical treatments although swallow dysfunction and other complications such as dryness and mucositis mean the optimal form of treatment remains a source of on-going debate [11–13]. The limitations of these treatment modalities have led some to explore whether tissue engineering could provide new therapeutic options.

Tissue engineering applies the principles of biological and material science with engineering to restore or replace damaged tissue or organs. The ability to transplant a functioning tissue-engineered larynx would revolutionize the treatment of laryngeal cancer by providing comparable rates of cure to conventional treatments whilst preserving function. Total cadaveric laryngeal transplantation has been reported twice in the literature and demonstrates the feasibility of total laryngeal replacement [14, 15]. A 14-year follow-up of the first transplant describes how after a 10-year period, a slow progressive chronic rejection process led to the organ becoming nonfunctional and the larynx being explanted [16]. It is possible that a tissue-engineered larynx would avoid this as it would be repopulated with the patient’s own cells. To date, reports on the delivery of a complete and functioning tissue-engineered larynx are however absent from the literature. The challenges involved in realizing this ambition are multiple and can be divided into challenges in scaffold design, re-epithelialization, and restoring laryngeal function.

## Scaffold Design of a Tissue-Engineered Larynx

A tissue-engineered total laryngeal replacement would require a scaffold that integrates with the host tissue, provides adequate structural support, and allows for mobility to enable laryngeal function. The first obvious target would be to engineer the larynx from the regenerated cartilage. Cartilage has the advantage of being biocompatible and exhibits a high tensile strength whilst retaining elasticity. The ability to engineer the cartilage in vitro has been extensively reported using a variety of protocols [17–20]. In brief, the cartilage is harvested from an autologous source such as the septum or auricle; the chondrocytes separated and expanded in vitro before reseeding onto a scaffold made from either biological, synthetic or biomimetic material. The successful integration in vivo of the engineered cartilage has remained problematic, however, with reports documenting strong inflammatory responses and rapid degradation as well as difficulties in restoring an adequate blood supply following transplantation [17, 21]. To overcome this, the principle of in vivo tissue engineering has been applied whereby the cartilage regenerates within a vascularized structure in vivo to enable transplantation with a preserved blood supply. An example of this can be seen in work by Kamil et al. where chondrocytes sourced from auricular cartilage were suspended in a biodegradable polymer and implanted in the dorsum of a pig [22]. After 2 months, a complete cartilage graft was harvested and used as part of a laryngotracheal reconstruction with bronchoscopy and histology demonstrating complete integration of the graft into the host tissue.

The use of synthetic or biomimetic scaffolds has the advantage over cartilage scaffolds in that they can be more easily fabricated to a specific size and shape and within a shorter period of time. Several studies have reported on the use of a polyprolene mesh coated with a spongy porcine collagen manufactured using a dental cast of a hemilarynx [23, 24]. Following hemilaryngectomy in a canine model, the construct is transplanted with a pre-clotted mixture of peripheral blood and bone marrow-derived stromal cells or a fascia lata wrap. After the first week, evidence of neo-mucosalization is demonstrated and the constructs had integrated within the host tissue.

Titanium has been used to reconstruct laryngotracheal resections for airway stenosis in a number of cases with the additional use of silk fibroin to facilitate better mucosal healing [25–28]. Titanium is an attractive synthetic material as it is hypoallergenic and comparatively cheap and has an extensive safety profile in other applications such as laryngeal fixation following fracture [29, 30]. Liu et al. used a titanium mesh to reconstruct laryngeal defects in nine patients following frontolateral vertical partial laryngectomy for T2 or T3 glottic cancer with the sternohyoid muscle being used to cover the mucosal surface of the mesh and the outer surface covered by omohyoid muscle [31]. No aspiration or laryngeal stenosis was observed following implantation and fiberoptic inspection showed the mesh remained covered in the long term.

Biological scaffolds can be fabricated by decellularizing donor tissue to remove the cellular content and thus immune potential whilst retaining the extracellular matrix proteomic cues that act as a map for regenerating cells [32]. Ansari et al. used a 6 × 4 × 2 m section of decellularized porcine larynx to replace a rectangular laryngeal defect in a pig model [33]. The scaffold was pre-implanted within a neck muscle flap for 4 weeks to re-vascularize before a sheet of engineered buccal mucosa was grafted onto the surface and the construct rotated on a muscle flap to repair the defect. Serial endoscopy and computer tomography imaging showed the graft integrated within this host tissue and a layer of neo-mucosa regenerated within the first month. None of the six grafts implanted led to any compromise in respiratory or swallowing function.

Aortic homografts are widely documented as a means of reconstructing tracheal defects and are available as “off the shelf” products that can be rapidly sourced and easily stored [34–38]. They also retain the extracellular matrix needed for successful regeneration and exhibit little immune response from the recipient [38–40]. Zeitels et al. used cryopreserved aortic homografts to reconstruct laryngeal defects in 15 patients undergoing partial laryngectomy for cancer [41]. The strap or sternocleidomastoid muscles were affixed to the extraluminal surface to promote revascularization, and a tracheostomy was sited in all patients to protect the graft in the initial post-operative phase. All of the 15 patients were successfully decannulated by 10 weeks and exhibited laryngeal phonation. Complete epithelialization took between 3 and 5 months and all but one of the patients resumed oral intake. Whilst this study demonstrates the success in using biological tissue to reconstruct partial defects, the subset of patients for which this type of resection is indicated is small and comparative studies with conventional reconstructive methods following partial laryngectomy are yet to be performed.

## Tissue Engineering to Restore Mucosal Function

Following scaffold selection, the means by which the mucosal layer will be restored needs to be determined. An intact mucosal layer is essential in any transplantation as it provides a barrier against infection. In laryngeal transplantation, this is even more relevant as the implant will be exposed to airborne pathogens during respiration. Mucosa also has a number of organ-specific specialized functions. Within the larynx, the lamina propria layer of the vocal folds needs to be sufficiently viscoelastic to allow for the transmission of the vibratory wave during phonation. Conventional methods of tissue replacement are not appropriate for restoring the mucosal layer in an engineered laryngeal transplant. Split skin grafts are associated with keratinization that leads to sloughing and infection within the airway, and buccal mucosal grafts are restricted by a limited supply of donor tissue. Myocutaneous flaps are ideal for reconstructing large defects but are too bulky to reline the complex three-dimensional surface of the larynx.

To achieve re-mucosalization of the laryngeal scaffold, several options are available.

The first is to rely on the migration of the host’s native epithelial cells to regenerate a new mucosal layer following implantation, a process that can be potentiated by the use of decellularized or biomimetic scaffolds that provide a favorable matrix environment for regenerating epithelium [42]. The delay in achieving re-mucosalization places that scaffold material at risk of infection, although this risk can be negated by the use of biological dressings, as seen with the use of a fascia lata flap and clotted peripheral blood in works by Yamashita and Kitani et al. [23, 24]. An alternative method might be to embed the mucosal surface of the scaffold with pro-migratory factors such as Rho-kinase inhibitor to potentiate the re-epithelialization process [43, 44].

**Fig. 1:**
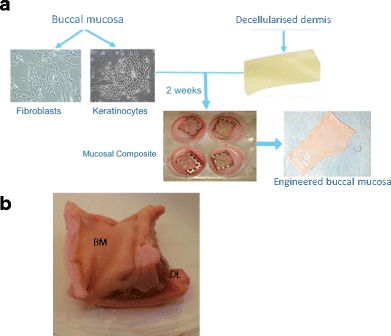
Tissue engineering buccal mucosa. **a** A biopsy of buccal mucosa is taken and the epithelial cells and fibroblasts separated. These cells are then expanded in culture and seeded onto a sheet of decellularized human dermis. The construct is then matured at air-liquid interface to promote differentiation. **b** A section of tissue-engineered buccal mucosa (*BM*) is sutured onto a decellularized hemilarynx (*DL*) in vitro to restore the mucosal layer in vitro

Another option to achieve re-mucosalization is to tissue engineer a separate mucosal layer and graft it onto the scaffold either before or during implantation. There are a number of examples demonstrating how stratified squamous mucosa can be engineered using autologous squamous cells expanded from a small biopsy and seeded onto decellularized dermis or collagen scaffolds (Fig. 1) [45, 46]. As with the engineered cartilage, difficulties arise in grafting the in vitro engineered mucosa onto a scaffold as, for the mucosa to survive, the scaffold requires a blood supply to deliver nutrition to the epithelial layer. Pre-vascularizing the scaffold within a muscle flap is a potential solution and allowed for the successful grafting of buccal mucosal grafts onto cadaveric trachea embedded within radial forearm fascia in a series of partial human tracheal transplants [47]. A similar method was employed in the study by Ansari et al. where a segment of decellularized porcine larynx was embedded within a neck muscle flap and engineered squamous mucosa grafted onto the surface 4 weeks later once a new blood supply had been restored [33]. In this instance, the mucosa did not appear to survive and acted as a biological dressing, protecting the scaffold, whilst the host’s native epithelial cells covered the defect. It could be argued that current examples of tissue-engineered mucosa are not suitable for relining complex three-dimensional scaffolds as the epithelial layer is exposed to shear during the implantation phase and there is a delay in re-vascularizing the epithelium following grafting leading to cell death. A potential solution could be to implant epithelial cells encased within collagen gel containing growth factors and/or mesenchymal cells that provide support to the epithelium whilst a blood supply is re-established. A similar technique has been used in gut engineering to reline a section of decellularized small intestine [48].

Studies aiming to restore the viscoelastic properties of the vocal fold lamina propria mainly involve the use of injectable gels such as synthetic hyaluronic acid-dextran [49], hyaluronic acid-based microgels [50], and collagen I composites [51]. These materials exhibit similar viscoelastic properties to healthy vocal fold lamina propria and are believed to be pro-regenerative leading to a restoration of a viscoelastic layer overtime [52]. The optimum material is still undecided however and whether a completely new lamina propria layer could be restored in an engineered vocal fold is yet to be determined as the majority of the studies examine the role of injectables in vocal fold scarring [53, 54].

## Tissue Engineering to Restore Dynamic Function

The larynx is a dynamic organ. During inspiration, the vocal folds abduct to allow airflow and during phonation, the folds adduct to generate vibration. During swallowing, there are a complex series of movements involving closure of the vocal and false cords, elevation of the larynx, and reflection of the epiglottis to prevent aspiration. A tissue-engineered total laryngeal transplant would need to be able to provide all of these functions if it is to overcome the shortcomings of conventional treatments. A partial laryngeal transplant might not require full function providing the cricoarytenoid-nerve-muscle unit is preserved in the un-resected larynx. The tissue-engineered partial implant would aim to improve upon the functional outcome of existing reconstructive techniques following partial laryngectomy, namely problems with aspiration.

The cricoarytenoid-nerve-muscle unit needs to be re-established in order to restore vocal fold movement. This would require the laryngeal scaffold to have a functioning cricoarytenoid joint, the successful regeneration and grafting of the cricoarytenoid muscles, and regeneration of the muscle’s nerve supply with appropriate central control. The success of conventional joint replacement limits the role of tissue engineering within this field to articular cartilage regeneration to treat damage to existing joints [55, 56]. Advances in nanoengineering might in the future deliver workable and biocompatible joint replacements small enough to restore cricoarytenoid movement but so far, examples of the application of this technology for this purpose are absent from the literature.

The use of tissue engineering techniques to regenerate the organized skeletal muscle has been well documented [57–59], although studies regenerating the cricoarytenoid muscle specifically are lacking. Fishman et al. demonstrated that rabbit cricoarytenoid dorsalis treated with latrunculin B, potassium iodide, potassium chloride, and deoxyribonuclease achieves total clearance of host DNA with preservation of the scaffold structural integrity and matrix components [60]. This provides a decellularized cricoarytenoid muscle that can act as a scaffold to regenerate a functioning cricoarytenoid muscle.

Reinnervation of the adductor and abductor cricoarytenoid muscles is currently performed to prevent vocal cord muscle atrophy and promote movement during respiration in cases of vocal fold paralysis. The procedure is performed by means of direct anastomosis of the adductor branch of recurrent laryngeal nerve with the ansa cervicalis and anastomosis of the abductor branch to the phrenic nerve [61–63]. Reports on functional outcome following reinnervation are promising [63–65] and demonstrate the feasibility of this technique to restore motor function in a tissue-engineered laryngeal transplant. The engineered cricoarytenoid muscle would either have to include neuromuscular endplates and a regenerated laryngeal nerve to anastomose onto the existing laryngeal nerve supply or the native laryngeal nerve would need to be anastomosed directly onto the regenerated cricoarytenoid muscle.

An alternative to reinnervation is the use of laryngeal pacing. Pacing involves the insertion of electrodes into the posterior cricoarytenoid muscle which deliver a time-phased electric signal that is in sync with the respiratory cycle to stimulate abduction of the vocal folds during inspiration [66]. This technology has been trailed in seven human patients with two of the seven subjects exhibiting full reanimation for greater than 4 years [67]. Problems with biocompatibility and the lack of a sensor to pace abduction with inspiration have meant this technology has not been widely adopted.

The restoration of swallowing function is more complex because of the need for a coordinated series of laryngeal movements. Transcutaneous neuromuscular electrical stimulation of the pharyngeal muscles has been used to bring about a coordinated muscle contraction in stroke patients suffering with dysphagia with promising results [68], and this technique could be adapted to reinnervate the larynx and coordinate the swallowing mechanism. Soft robotics aims to deliver robotic devices that are flexible and can potentially be used as implants. This could deliver fully automated laryngeal movement to restore swallow and voice although the technology is still in the early development phases [69, 70]. A more rudimentary example of a mechanical larynx has been trialed using a titanium prosthesis with a concentric valve system that opens to allow respiration and closes to avoid aspiration during swallowing. Pre-clinical studies were encouraging in terms of prosthetic integration [71, 72], although in the single human case reported, the patient was still tracheostomy dependent and due to the absence of a glottic sphincter phonation was poor [73]. Long-term follow-up of this case and a clinical trial involving this technology has not yet been published.

## Conclusions

There is great potential for tissue engineering to revolutionize the treatment of laryngeal cancer in the form of a partial or total laryngeal transplant within the next 10 years. A number of challenges remain which are best addressed by a coordinated effort involving biological and material scientists, engineers, surgeons, and allied health professionals. A decision on the choice of scaffold and the optimal method of achieving integration with revascularization should form any early milestone and should be developed in continuity with methods of achieving complete re-mucosalization. Without function, total laryngeal replacement would not be able to greatly improve upon existing treatments for laryngeal cancer. Developments in restoring laryngeal function must therefore keep pace with developments in laryngeal scaffold and mucosal engineering.
